# Designing a strategy to implement cost-effective blood transfusion management in elective hip and knee arthroplasties: A study protocol

**DOI:** 10.1186/1748-5908-7-58

**Published:** 2012-06-30

**Authors:** Veronique MA Voorn, Perla J Marang-van de Mheen, Cynthia So-Osman, Thea PM Vliet Vlieland, Ankie WMM Koopman-van Gemert, Rob GHH Nelissen, Leti van Bodegom-Vos

**Affiliations:** 1 Department of Medical Decision Making, Leiden University Medical Centre, Leiden, The Netherlands; 2 Jon J van Rood Netherlands Center for Clinical Transfusion Research, Sanquin Research, Leiden, The Netherlands; 3 Department of Orthopedics, Leiden University Medical Centre, Leiden, The Netherlands; 4 Department of Anesthesiology, Albert Schweitzer Hospital, Dordrecht, The Netherlands

**Keywords:** Hip/Knee arthroplasties, Blood transfusion, Blood-saving measures, Problem analysis, Barriers and facilitators, Implementation strategy

## Abstract

**Background:**

Total hip and knee arthroplasties are two of the most commonly performed procedures in orthopedic surgery. Different blood-saving measures (BSMs) are used to reduce the often-needed allogenic blood transfusions in these procedures. A recent large randomized controlled trial showed it is not cost effective to use the BSMs of erythropoietin and perioperative autologous blood salvage in elective primary hip and knee arthroplasties. Despite dissemination of these study results, medical professionals keep using these BSMs. To actually change practice, an implementation strategy is needed that is based on a good understanding of target groups and settings and the psychological constructs that predict behavior of medical professionals. However, detailed insight into these issuses is lacking. Therefore, this study aims to explore which groups of professionals should be targeted at which settings, as well as relevant barriers and facilitators that should be taken into acount in the strategy to implement evidence-based, cost-effective blood transfusion management and to de-implement BSMs.

**Methods:**

The study consists of three phases. First, a questionnaire survey among all Dutch orthopedic hospital departments and independent treatment centers (n = 99) will be conducted to analyze current blood management practice. Second, semistructured interviews will be held among 10 orthopedic surgeons and 10 anesthesiologists to identify barriers and facilitators that are relevant for the uptake of cost-effective blood transfusion management. Interview questions will be based on the Theoretical Domains Interview framework. The interviews will be followed by a questionnaire survey among 800 medical professionals in orthopedics and anesthesiology (400 professionals per discipline) in which the identified barriers and facilitators will be ranked by frequency and importance. Finally, an implementation strategy will be developed based on the results from the previous phases, using principles of intervention mapping and an expert panel.

**Discussion:**

The developed strategy for cost-effective blood transfusion management by de-implementing BSMs is likely to reduce costs for elective hip and knee arthroplasties. In addition, this study will lead to generalized knowledge regarding relevant factors for the de-implementation of non-cost-effective interventions and insight in the differences between implementation and de-implementation strategies.

## Background

Total hip and knee arthroplasties are two of the most commonly performed procedures in orthopedic surgery [1,2]. It is expected that the number of these procedures within the Netherlands will increase to more than 100,000 by the year 2030 [3]. During primary hip or knee arthroplasty, the calculated visible and invisible blood loss is 1,500 ml on average, followed by a drop of hemoglobin of approximately 3 g/dl [4]. This leads to high rates of allogenic blood transfusions up to 69% depending on the transfusion threshold [5]. Even though blood transfusions may be necessary, they include the risk for infections and noninfectious transfusion reactions [6].

Many studies on blood-saving measures (BSMs) have therefore been performed, including erythropoietin (EPO) and peri-operative autologous blood salvage (intra-operative use of Cell Saver (CS) and a postoperative drainage and reinfusion device (DR)). Reviews showed that these studies had several limitations, such as a retrospective design, small patient numbers and poor methodological quality [5,7,8]. A multicenter randomized controlled trial (RCT) with adequate power (n = 2,442) was therefore performed recently to test the cost effectiveness of using BSMs, including EPO, CS, and DR, in elective primary hip and knee arthroplasties[9]. It was shown that blood salvage (CS and DR) resulted in neither decreased mean red blood cell (RBC) use nor in a decrease in the proportion of transfused patients and was more expensive due to the costs of the devices used and a prolonged hospital stay. EPO showed a significant decrease in the proportion of transfused patients, but costs were considered too high. It was thus concluded that these BSMs were not cost effective in primary hip and knee arthroplasties [10].

Despite this evidence about BSMs not being cost effective, medical professionals keep using these BSMs in daily practice. To decrease costs of care delivery to patients undergoing a hip or knee arthroplasty, cost-effective blood transfusion management needs to be implemented. However, little is known about how to effectively de-implement common practices. To actually change practice, a de-implementation strategy is needed that is based on a good understanding of target groups and settings and the barriers and facilitators that influence the behavior of medical professionals. [10,11] However, detailed insight into these factors is lacking. Psychological theories are used in understanding and predicting intentions and clinical behavior [12] and may help to outline an effective strategy to de-implement these non-cost-effective BSMs.

## Objective

The Leiden Implementation Study of BlOod management in hip and knee Arthroplasties (LISBOA) aims to explore the target groups, settings, and relevant barriers and facilitators that should be taken into account to develop a strategy directed at all involved medical professionals (target group) and their organizations to implement evidence-based, cost effective transfusion management and to de-implement BSMs.

To reach the aim of this study, the following research questions were formulated:

A. How often and in what settings are BSMs applied in hip and knee arthroplasties?

B. Which barriers and facilitators influence the implementation of cost-effective blood transfusion management and de-implementation of non-cost-effective BSMs among the target group, including orthopedic surgeons and anesthesiologists?

C. What is a tailored implementation strategy for the uptake of cost-effective blood transfusion management given the results of the first two research questions?

## Methods

The study will be subdivided in three study phases to be executed in one year:

A. analysis of current blood transfusion management practice in elective primary hip and knee arthroplasties (months 1 to 3),

B. analysis of barriers and facilitators relevant for the implementation of cost-effective blood transfusion management and de-implementation of non-cost-effective BSMs (months 4 to 8),

C. development of an implementation strategy based on the results of phases A and B (months 9 to 12).

The study design, study population, analysis, and outcome measures are described per study phase.

## Phase A: Analysis of current blood transfusion management

### Study design

To analyze current blood transfusion management practice in hip and knee arthroplasties, a survey among all orthopedic departments of Dutch university, teaching, and general hospitals and independent treatment centers will be performed. A survey in the period 1995–1997 showed that EPO was used rarely in the Netherlands at that time, in only 2% of all hospitals, and that CS was used in 24% of hospitals [13]. A more recent survey in 2007 showed that approximately half of all Dutch orthopedic departments applied EPO and/or autologous blood salvage [14]. However, these surveys neither showed how frequent these BSMs were applied within hospitals nor in what type of setting (university, teaching, or general hospital or independent treatment center). This information is needed to target the implementation strategy to the appropriate professionals and departments.

The current survey will thus include questions about the type and size of the department, the transfusion protocol used, and the frequency of application of BSMs in patients within the last 12 months. Furthermore, questions will be included about the policy of preoperative anticoagulant use. These last questions are added to assess whether these protocols are related to BSM use and should be taken into account in the implementation strategy. The content of the survey will be developed together with an orthopedic surgeon, anesthesiologist, and hematologist specialized in blood transfusions. Reminders to nonresponders will be sent after two weeks and again by telephone after four weeks.

### Study population

All heads of orthopedic departments of Dutch university, teaching, and general hospitals and independent treatment centers (n = 99) will be approached to participate in the survey. In case of nonresponse, a different orthopedic surgeon within the same department will be approached.

### Analysis

Descriptive statistics will be used to describe current blood management practice. Independent *t* tests or Mann Whitney U tests for continuous variables and Chi-Square tests or Fisher’s exact tests for proportions are used to analyze differences in frequency of use between the different settings, department sizes, or other conditions.

### Outcome measures

The main outcome measures are the percentage of orthopedic departments applying BSMs per size and type of setting of the orthopedic department and the frequency of BSM use within a department. These results are used in phase C to address the implementation strategy to the appropriate (groups of) orthopedic departments. A secondary outcome measure is the number of days anticoagulants are stopped preoperatively. This is used to analyze whether this is associated with BSM use and should be taken into account in the implementation strategy.

## Phase B: Analysis of barriers and facilitators for implementation of cost-effective blood transfusion management

### Study design

Two steps will be taken to identify barriers and facilitators associated with the implementation of cost-effective blood transfusion management. First, semistructured interviews will be performed to explore all relevant barriers and facilitators for the uptake of cost-effective blood transfusion management. The interview questions will be based on the Theoretical Domains Interview (TDI) framework [15], complemented by the framework of Cabana, who subdivided largely similar constructs in three “sequences of behavior change” to give a good overview of the used constructs [16]. The TDI framework includes 12 theoretical construct domains derived from 33 health psychology theories (covering 128 theoretical constructs) that help explain clinicians’ behavior [15,17].

Second, a survey will be held among a random sample of 400 Dutch orthopedic surgeons and 400 anesthesiologists to rank the barriers and facilitators identified in the interviews both on frequency and importance. The survey will include questions in which these barriers and facilitators of the identified theoretical domains can be related to specific clinical behavior.

### Study population

Orthopedic surgeons and anesthesiologists are key stakeholders in deciding to use allogenic blood transfusions only or BSMs in patients that undergo hip and knee arthroplasty. Based on the analysis of current practice (phase A of this study), we will select a sample of departments that frequently apply BSMs to identify barriers, as well as departments with rare use of BSMs to identify facilitators. In this selection, the setting is taken into account (university, teaching, or general hospital or independent treatment center). In addition, departments with alternative answers (*e.g.*, the use of a different transfusion protocol) will be selected for interviews. In total, ten orthopedic surgeons and ten anesthesiologists will be interviewed to identify barriers and facilitators relevant for the uptake of a cost-effective blood transfusion policy and their motivations to apply BSMs. Data saturation for the interviews is defined as three consecutive interviews without new themes emerging. If there is no data saturation after 10 interviews per specialism, additional interviews will be conducted. [18] The total number of interviews will thus be determined by the number it takes to reach data saturation.

The interviews with orthopedic surgeons and anesthesiologists may reveal that other groups of stakeholders have an important role in deciding to use BSMs. In that case, additional interviews will be held with those stakeholders to elicit their views about relevant barriers and facilitators associated with the uptake of cost-effective transfusion management.

For the survey, a random sample (n = 400) of all Dutch orthopedic surgeons listed in the registry of the Dutch Orthopedic Association (NOV) (n = 595) and a random sample (n = 400) of anesthesiologists listed in the registry of the Netherlands Society of Anesthesiologists (NVA) (n ≈ 1200) will be approached for participation in the survey.

### Analysis

The interviews will be audiotaped and transcribed in full for analysis. The interview transcripts will be analyzed by two researchers using the TDI framework as a base [15]. Important theoretical domains and the barriers and facilitators within these domains will be coded. This qualitative analysis will be executed using the software package ATLAS.ti (ATLAS.ti Scientific Software Development GmBH, Berlin, Germany).

The subsequent survey data will allow us to rank the importance of barriers and facilitators and their relationships with behavioral intention. These relationships will be assessed using regression analysis.

### Outcome measures

The most important barriers and facilitators relevant for the uptake of cost-effective blood transfusion management by medical professionals will be the outcome measures from this phase.

## Phase C: Development of an effective implementation strategy for cost-effective blood management

### Study design

The results from the previous phases will be used to develop a tailored implementation strategy for cost-effective blood transfusion management for elective primary hip and knee arthroplasties. The results from phase A will show to which type of departments the strategy should be aimed. Phase B results will show the most important barriers and facilitators that should be taken into account in the development of the strategy.

From the literature, it is known that, in general, multifaceted strategies are more effective than single strategies [19,20]. Assuming this, and our expectation that several barriers on different theoretical domains will be found, it is very likely that the implementation strategy to be developed will include several components directed at different levels (*i.e.*, professional and organizational context). Furthermore, it is expected that the strategy components will include educational outreach or interactive educational strategy since these are known to be effective [20,21].

In the development process, we will use a method based on the intervention mapping approach of Bartholomew *et al.*[22]. This method begins with the creation of matrices in which the performance objectives are set against the top 10 ranking of factors that hinder or facilitate the implementation of a cost-effective transfusion policy. Subsequently, a brainstorming session will be held about the strategy components needed to achieve the performance objective, in the presence or absence of the hindering or facilitating factor mentioned in the matrix. The cells of the matrices will then gradually be filled with implementation strategy components [23]. Next, the formulated strategy components will be translated into practical strategies at each level (*e.g.*, professional and organizational).

After the implementation strategy is developed, an expert meeting will be held with a panel of key opinion leaders in orthopedic surgery and anesthesiology, delegates of blood transfusion committees, and implementation experts (n = 10 to n = 20) to discuss the strategy’s feasibility and to refine the developed implementation strategy. Their opinion about the strategy and their intention to use the strategy will be taken into account.

### Analysis

The expert meeting will be audiotaped and transcribed. The panel members will receive a summary of the formulated implementation strategy and will be asked whether this summary is consistent with the conclusions reached in the meeting.

### Outcome measures

The outcome from this phase will be a tailored implementation strategy likely to be effective for implementing cost effective blood transfusion management and de-implementing BSMs in elective primary hip and knee athroplasties.

## Ethical approval

The study protocol has been presented to the Medical Ethical Committee of the Leiden University Medical Center. They declared ethical approval was not required under Dutch national law. (CME 11/104)

## Discussion

The goal of this study is to develop an implementation strategy for cost-effective blood transfusion management in elective hip and knee arthroplasties in which BSMs are de-implemented. This study is the next step following a RCT on EPO and blood salvage as transfusion alternatives in orthopedic surgery using a restrictive transfusion policy that showed that use of these BSMs is not cost effective [9]. Given the number of hip and knee arthroplasties performed annually in the Netherlands and worldwide, and the accompanied blood loss and transfusion risks, implementing a cost-effective blood transfusion management may reduce costs.

Several studies have been performed to develop and test implementation strategies, including identification of barriers that prevent implementation [10,16,19]. They all conclude that a prior inventory of barriers to develop a tailored implementation strategy is useful and can confirm whether barriers differ in different settings. Prior inventory thereby reduces the number of costly trials evaluating different implementation strategies. [11,24,25] The present study, however, focuses on de-implementation of BSMs known to be cost ineffective. Little is known about barriers and facilitators for de-implementation and whether these are similar to barriers and facilitators for implementation. The knowledge obtained by the present study may thus be further generalized to other practices that need to be de-implemented and contributes to general knowledge regarding differences between de-implementation and implementation strategies.

## Strengths and limitations

Possible limitations of the study are biased results due to response bias in the phase A survey [26]. Nonresponse may cause an under- or overestimation of BSM use. The selection for the interviews in phase B is based on the results of phase A, so if non-responders have different intentions or experience different barriers and facilitators for the uptake of cost-effective blood transfusion management, this may influence the resulting barriers and facilitators.

We will try to overcome this by sending reminders by email and telephone, but this will not completely prevent response bias. In addition, response bias may also occur in the phase B survey if nonresponders to this survey rank the selected barriers and facilitators in a different order; this may influence the likelihood of barriers and facilitators being included in the implementation strategy. Again, reminders will be sent to keep bias to a minimum, and we will compare respondents and nonrespondents on demographic variables (*e.g.*, type of hospital) to estimate how likely it is that bias may be introduced.

A strength of this study is that it is one of the first studies to identify barriers and facilitators relevant for de-implementation. The study results will thus lead to generalized knowledge regarding factors that are important for the de-implementation of non-cost-effective interventions and how these differ from relevant factors for implementation.

## Future work

The developed implementation strategy should be tested for effectiveness, feasibility, and costs within orthopedic practice in the Netherlands in a future study. As the current implementation strategy will be aimed at de-implementation of the use of EPO, CS, and DR, further research is needed to evaluate the cost effectiveness of other BSMs in hip and knee arthroplasties. Cost effective blood transfusion management implemented in this way is likely to improve efficiency of care.

## Competing interests

The authors have no competing interests to declare.

## Authors’ contributions

LB, AK, RN, CS, and TV designed the study; VV will carry out the study; LB and PM will coordinate the study. VV, LB, and PM drafted the manuscript. The manuscript has been read and approved by all authors.
